# Systematic Comparison of Left Ventricular Geometry Between 3D-Echocardiography and Cardiac Magnetic Resonance Imaging

**DOI:** 10.3389/fcvm.2021.728205

**Published:** 2021-09-20

**Authors:** Debbie Zhao, Gina M. Quill, Kathleen Gilbert, Vicky Y. Wang, Helene C. Houle, Malcolm E. Legget, Peter N. Ruygrok, Robert N. Doughty, João Pedrosa, Jan D'hooge, Alistair A. Young, Martyn P. Nash

**Affiliations:** ^1^Auckland Bioengineering Institute, University of Auckland, Auckland, New Zealand; ^2^Siemens Healthineers, Issaquah, WA, United States; ^3^Department of Medicine, University of Auckland, Auckland, New Zealand; ^4^Green Lane Cardiovascular Service, Auckland City Hospital, Auckland, New Zealand; ^5^Institute for Systems and Computer Engineering, Technology and Science, Porto, Portugal; ^6^Department of Cardiovascular Sciences, KU Leuven, Leuven, Belgium; ^7^Department of Biomedical Engineering, King's College London, London, United Kingdom; ^8^Department of Anatomy and Medical Imaging, University of Auckland, Auckland, New Zealand; ^9^Department of Engineering Science, University of Auckland, Auckland, New Zealand

**Keywords:** 3D-echocardiography, cardiac MRI, left ventricle, image registration, reproducibility

## Abstract

**Aims:** Left ventricular (LV) volumes estimated using three-dimensional echocardiography (3D-echo) have been reported to be smaller than those measured using cardiac magnetic resonance (CMR) imaging, but the underlying causes are not well-understood. We investigated differences in regional LV anatomy derived from these modalities and related subsequent findings to image characteristics.

**Methods and Results:** Seventy participants (18 patients and 52 healthy participants) were imaged with 3D-echo and CMR (<1 h apart). Three-dimensional left ventricular models were constructed at end-diastole (ED) and end-systole (ES) from both modalities using previously validated software, enabling the fusion of CMR with 3D-echo by rigid registration. Regional differences were evaluated as mean surface distances for each of the 17 American Heart Association segments, and by comparing contours superimposed on images from each modality. In comparison to CMR-derived models, 3D-echo models underestimated LV end-diastolic volume (EDV) by −16 ± 22, −1 ± 25, and −18 ± 24 ml across three independent analysis methods. Average surface distance errors were largest in the basal-anterolateral segment (11–15 mm) and smallest in the mid-inferoseptal segment (6 mm). Larger errors were associated with signal dropout in anterior regions and the appearance of trabeculae at the lateral wall.

**Conclusions:** Fusion of CMR and 3D-echo provides insight into the causes of volume underestimation by 3D-echo. Systematic signal dropout and differences in appearances of trabeculae lead to discrepancies in the delineation of LV geometry at anterior and lateral regions. A better understanding of error sources across modalities may improve correlation of clinical indices between 3D-echo and CMR.

## Introduction

Echocardiography (echo) is the most ubiquitous cardiovascular imaging modality, with applications both at bedside and during intervention. Although traditionally a two-dimensional (2D) modality, three-dimensional echocardiography (3D-echo) enables analyses of left ventricle (LV) structure and function without the need for geometric assumptions. Furthermore, 3D models of the LV can be used to precisely quantify remodeling (1) and calculate biophysical properties such as myocardial stiffness (2).

The traditional validation approach for 3D-echo-derived LV models has involved direct comparisons of volumetric indices against cardiac magnetic resonance (CMR)-derived reference values (3), which has been shown to provide a precise estimation of LV geometry (4). Although 3D-echo is known to underestimate LV volume compared to CMR (5), the reasons for this discrepancy are not well understood. Moreover, existing studies are typically focused on global measurements and thus do not account for regional differences, with some exceptions (6–8).

In a controlled comparison of 3D-echo and CMR-derived end-diastolic volumes (EDV) in a small group of children with hypoplastic left heart syndrome (9), Gomez et al. identified regional differences in geometry due to disparities in image appearance between the two modalities. Here, we sought to extend this approach to investigate the differences between 3D-echo and CMR in a mixed population of healthy participants and patients with various acquired cardiac diseases. Subsequently, LV geometric differences at end-diastole (ED) and end-systole (ES) were quantified in terms of regional surface distances with respect to the American Heart Association (AHA) 17-segment model (10).

While variability measures can be readily assessed for data from a single modality, comparisons between different modalities are complicated by the prerequisite of an image alignment step. Therefore, we employed data fusion using geometry registration to provide a regional comparison between 3D-echo and CMR, which enabled the identification of potential sources of error when extracting LV geometries from 3D-echo. To examine inter-software variability, we compared results from three software solutions currently available for 3D-echo image analysis. Finally, we discuss how fusion of images between modalities can potentially aid operator training and suggest ways to improve current analysis routines.

## Materials and Methods

### Study Design and Data Acquisition

Seventy participants (47 male and 23 female; 52 healthy participants with no known cardiovascular condition, 10 patients with LV hypertrophy, 8 patients with aortic regurgitation) were prospectively recruited for non-invasive imaging under 3D-echo and CMR (<1 h apart). Ethical approval for this study was granted by the Health and Disability Ethics Committee of New Zealand (17/CEN/226), and written informed consent was obtained from each participant.

Real-time transthoracic 3D-echo acquisitions of the LV was performed using a Siemens ACUSON SC2000 Ultrasound System with a 4Z1c transducer (Siemens Medical Solutions, Mountain View, CA, USA) from the apical window in a left lateral decubitus position. Echo acquisition parameters were optimized for each subject (resulting in an average of 36 echo image frames per cardiac cycle). Cine CMR imaging was performed on either a Siemens Magnetom 1.5T Avanto Fit (35 male; 12 female) or 3T Skyra (12 male; 11 female) (Siemens Healthcare, Erlangen, Germany). Images were acquired in three long-axis slices (two-, three-, and four-chamber views) and six equally spaced short-axis slices (spanning from the LV apex to the mitral valve, with an average slice gap of 18 mm) using a balanced steady-state free precession sequence with the following typical imaging parameters: TR = 3.7 ms, TE = 1.6 ms, flip angle = 45°, field of view (FOV) = 360 × 360 mm, isotropic pixels of 1.4 × 1.4 mm, and slice thickness = 6 mm. By fixing the number of short-axis slices and varying the slice gap, sampling frequency in the long-axis direction was therefore maintained relative to LV size across subjects. With these parameters, an average of 30 CMR image frames were acquired per cardiac cycle across the study population.

### Image Analysis

To minimize performance bias (e.g., caused by the consecutive analysis of the same subject across modalities and software), analysis of CMR and 3D-echo was performed separately, such that analysis using a new method commenced only after analysis of the entire cohort had been completed by the preceding method, with the analyses performed at least 3 weeks apart.

**Figure 1:**
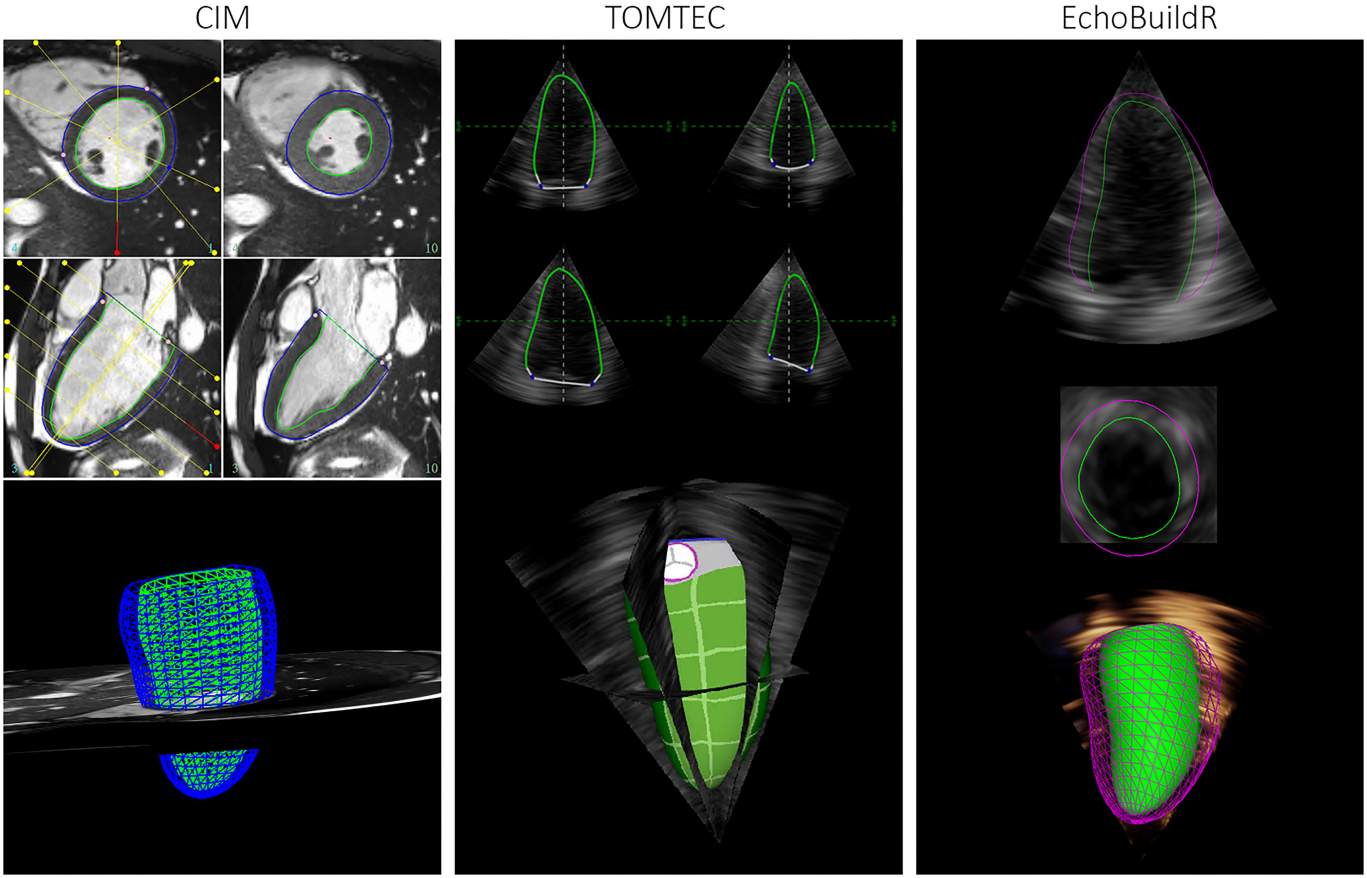
Graphical user interface for semi-automatic construction of LV geometries in CIM **(left)**, TOMTEC **(middle)**, and EchoBuildR **(right)**. (Note that dynamic epicardial modeling was not supported in TOMTEC).

Cardiac magnetic resonance data analysis was performed offline using Cardiac Image Modeller (CIM, v8.1, University of Auckland, New Zealand), a validated semi-automatic software tool based on a geometric finite element (FE) model of the LV (11). For all 70 subjects, the same analyst (Expert A, experienced in the analysis of both CMR and 3D-echo) identified four types of fiducial landmarks (i.e., valve inserts at the base of the LV myocardium, apical centroid, basal centroid, and right ventricular (RV) insertion points along the LV epicardial border), applied corrections of in-plane breath-hold mis-registrations, and interactively fit contours to the endocardium and epicardium. This was repeated on each slice plane (see Figure 1) over one cardiac cycle. Papillary muscles and trabeculations were excluded from the myocardium. Although the inter-observer variability for CMR is typically small (12), this was quantified for the present study with a second observer (Expert B, experienced in CMR analysis), who independently performed full CMR analyses (from landmark identification to contour fitting) for a subset of 20 subjects.

To investigate inter-software variability in 3D-echo LV geometric analysis, three different tools were selected. Two were semi-automatic methods—TOMTEC 4D LV-ANALYSIS 3 (TOMTEC Imaging Systems GmbH, Unterschleissheim, Germany), a commercially available echo analysis software suite; and EchoBuildR 3.4.0 (Siemens Medical Solutions, Mountain View, CA, USA) prototype software based on boundary detectors and a statistical shape model constructed from a large expert-annotated database (13). Left ventricular models were created by interactively manipulating the contours to fit the myocardium, carried out by the same observer (Expert A) in both applications (Figure 1) on separate occasions, after having completed CMR analysis for all participants. To estimate the inter-observer variability associated with 3D-echo, a third observer (Expert C, an experienced clinical cardiac sonographer) repeated the geometric analysis on all 70 subjects using the TOMTEC application.

Finally, a previously validated fully automated approach based on a B-spline Explicit Active Surfaces (BEAS) algorithm (14), having outperformed several other state-of-the-art methods at the MICCAI 2014 CETUS challenge (15), was used to provide an observer-independent segmentation of the 3D-echo dataset.

### Fusion of 3D-Echo and CMR

In general, the registration of images across distinct modalities is not trivial due to inconsistency in image characteristics. For 3D-echo, this is further exacerbated by acoustic shadowing and variation in appearances of the same tissue structure depending on the angle of incidence of the ultrasound beam.

To circumvent these challenges, the alignment of 3D-echo and CMR was carried out by rigid registration of the LV landmarks and surfaces into a common cardiac coordinate system. For CMR, this was defined by a central axis connecting the apical and basal centroids, with an RV centroid (calculated as the mean position of all RV insertion points) used to orient each LV model about its long-axis. For 3D-echo, apical and basal centroids were computed from the output of the TOMTEC, EchoBuildR, and BEAS algorithms, following which an RV centroid was approximated as being 70 degrees clockwise from the inferior RV insertion when viewed from apex to base (as the anterior RV insertion generally could not be seen in 3D-echo). Finally, the LV FE model surfaces were re-fitted using least squares minimization to the 3D-echo endocardial and epicardial surface points (as exported from each of the 3D-echo segmentation tools). By registering both CMR and 3D-echo models to this common cardiac coordinate system, an initial coarse registration was consequently achieved by aligning the LV long-axis and RV direction.

**Figure 2:**
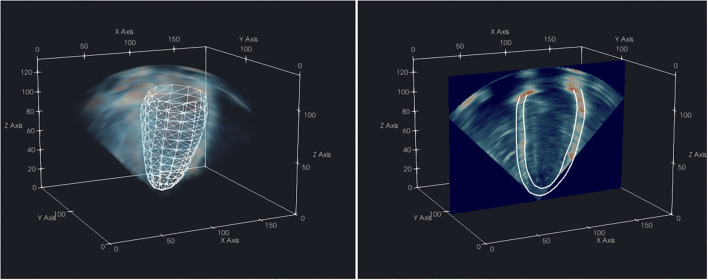
Visualization of a CMR-derived LV endocardial surface mesh aligned with the corresponding 3D-echo volume **(left)** from the same subject, and 2D slice with resultant endocardium and epicardium contours **(right)** in ParaView 5.8.0.

The coarse alignment between the CMR FE mesh and 3D-echo was subsequently refined by manually applying translations and rotations in ParaView 5.8.0 (16) (Figure 2), with visualization examples provided in the Supplementary Videos 1,2. These were conducted independently for image frames corresponding to ED and ES, as slight changes in transducer position relative to the heart could occur during acquisition. Once verified by visual inspection (conducted by Expert A), the series of rigid transformations were applied to align the CMR images and model with the corresponding 3D-echo image.

### Regional Surface Distances

Left ventricular geometries were divided into regional segments as detailed in the work of Chan et al. (17). Differences between re-fitted 3D-echo and CMR geometries were expressed as mean surface distances (MSD), separately for each of the 16 AHA segments of the endocardium (excluding the apical cap) and 17 AHA segments of the epicardium, computed as:


$$\begin{array}{l} MSD_{AHA}=\;\frac{1}{N_{p}}\left(\overset{N_{p}}{\underset{p=1}{\sum }}d\left(M_{p}\;,\;E_{p}\right)\right) \end{array}$$

Here, *d*(*M*_*p*_, *E*_*p*_) represents the Euclidean distance between the corresponding points *M*_*p*_ from the CMR model and *E*_*p*_ from the re-fitted 3D-echo model; and *N*_*p*_ represents the number of surface points (which were variable between regions) within a particular AHA segment. This method enabled an equivalent representation of LV geometry between CMR and 3D-echo to allow for direct comparisons on a regional basis.

### Statistics

A paired sample *t*-test was performed between CMR-derived volumetric measurements and 3D-echo-derived measurements for each of the three 3D-echo segmentation methods. A two-tailed *P* < 0.0167 (after applying the Bonferroni correction for multiple comparisons) was considered statistically significant.

An average measure intraclass correlation coefficient (ICC) based on a two-way mixed-effects model was independently calculated for each measured index to assess the degree of absolute agreement between corresponding methods.

## Results

### CMR to 3D-Echo Alignment

**Figure 3:**
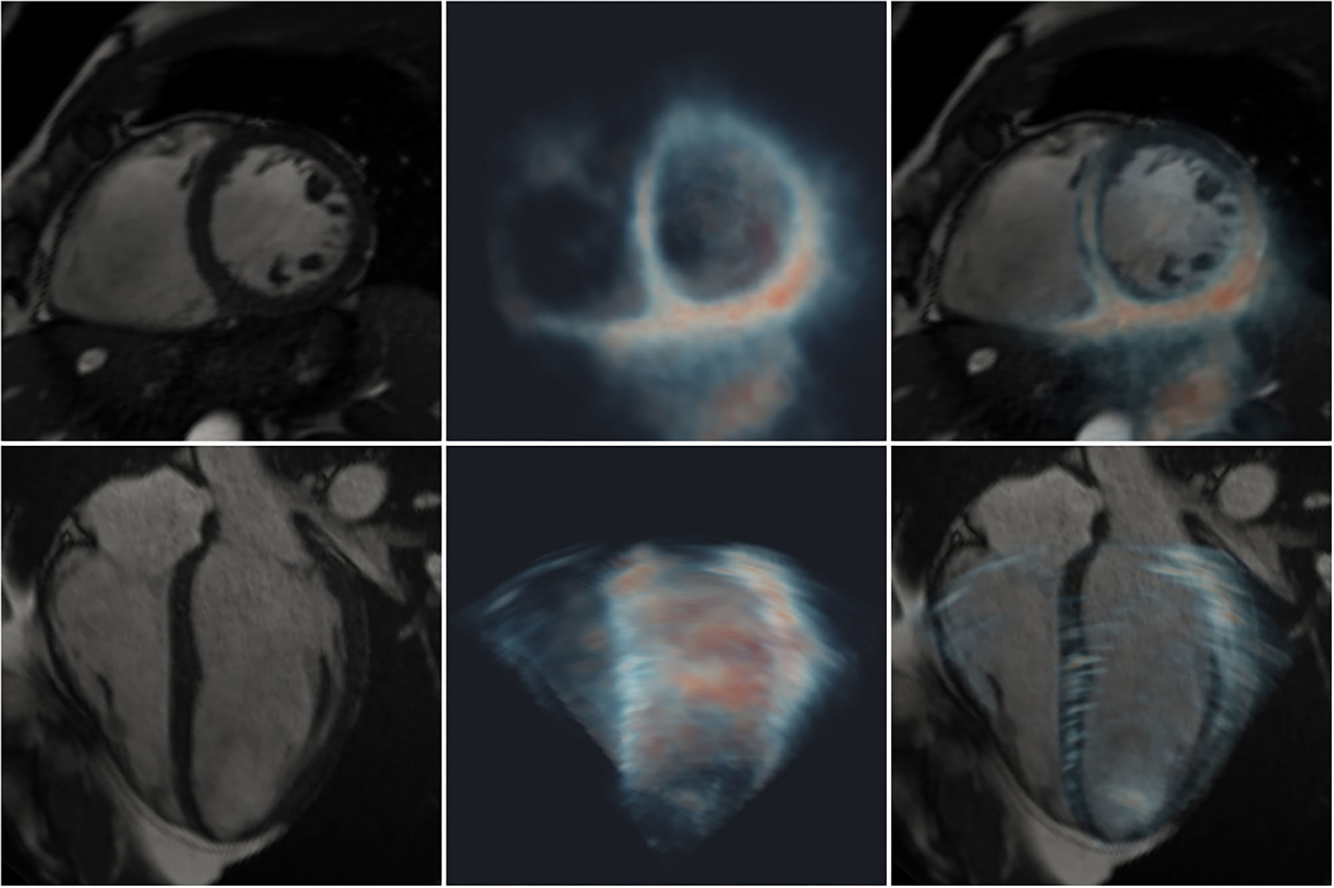
Registration of 2D CMR images (greyscale) with a 3D-echo image volume (maximum intensity projection using blue-to-red colormap). Short- **(top row)** and long- **(bottom row)** axis views are shown. **Left:** CMR; **middle:** 3D-echo; **right:** fused CMR and 3D-echo.

All cases exhibited good visual alignment of CMR and 3D-echo using the interactive rigid registration method. An example is shown in Figure 3, where variable signal intensity is seen across the 3D-echo image. Further examples showing the alignment at different stages during the cardiac cycle for additional subjects can be found in the Supplementary Videos 3–5.

### Differences in Global LV Indices

Differences in global LV measurements and ICCs are presented in Table 1. The ages of the participants ranged between 18 and 79 years (mean ± standard deviation (SD) of 41 ± 20 years), and body surface area ranged between 1.41 and 2.37 m^2^ (1.88 ± 0.22 m^2^).

**Table 1:** Differences in global LV indices (3D-echo–CMR, presented as mean ± SD) across 70 subjects for each 3D-echo segmentation tool compared to CMR-derived values with corresponding intraclass correlation coefficients (ICC).

	**CMR**	**TOMTEC**		**EchoBuildR**		**BEAS**	
		**Difference**	**ICC**	**Difference**	**ICC**	**Difference**	**ICC**
EDV (ml)	150 ± 36	−16 ± 22*	0.878	−11 ± 25*	0.851	−18 ± 24*	0.831
EDVI (ml/m^2^)	80 ± 16	−8 ± 12*	0.831	−6 ± 13*	0.778	−10 ± 13*	0.736
ESV (ml)	56 ± 21	−2 ± 14	0.880	−1 ± 15	0.845	9 ± 18*	0.771
ESVI (ml/m^2^)	30 ± 11	−1 ± 7	0.873	0 ± 8	0.832	5 ± 9*	0.753
EF (%)	63 ± 6	−3 ± 6*	*N/A*	−3 ± 6*	*N/A*	−12 ± 8*	*N/A*
LVM (g)	135 ± 41	*N/A*		−1 ± 28	0.841	−13 ± 38*	0.667
LVMI (g/m^2^)	71 ± 18			0 ± 15	0.728	−7 ± 21*	0.420

Asterisks (*) indicate statistically significant differences between 3D-echo and CMR. (Note that ICCs were not calculated for EF as it is derived from EDV and ESV, and is not a direct measurement).

Significant differences in EDV and end-diastolic volume index (EDVI) were found between CMR and 3D-echo for all three software tools (all *P* < 0.0005), with 3D-echo EDV being smaller than the CMR EDV. No significant differences in end-systolic volume (ESV) (*P* = 0.228, 0.763 for TOMTEC and EchoBuildR, respectively) and end-systolic volume index (ESVI) (*P* = 0.159, 0.686 for TOMTEC and EchoBuildR, respectively) were found between CMR and 3D-echo for the two semi-automatic methods, but BEAS gave rise to a significantly larger ESV compared to that of CMR (*P* = 0.0001 for both ESV and ESVI). Further significant differences were found between CMR and 3D-echo in terms of ejection fraction (EF) (all *P* < 0.0001). For left ventricular mass (LVM) and left ventricular mass index (LVMI) (calculated from the ED models), no significant difference was found between CMR and EchoBuildR (*P* = 0.819, 0.971 for LVM and LVMI, respectively), but BEAS yielded a significantly lower LVM (*P* = 0.006) and LVMI (*P* = 0.011). LVM was not obtained by TOMTEC.

### Surface Distances Between 3D-Echo and CMR Models

**Figure 4:**
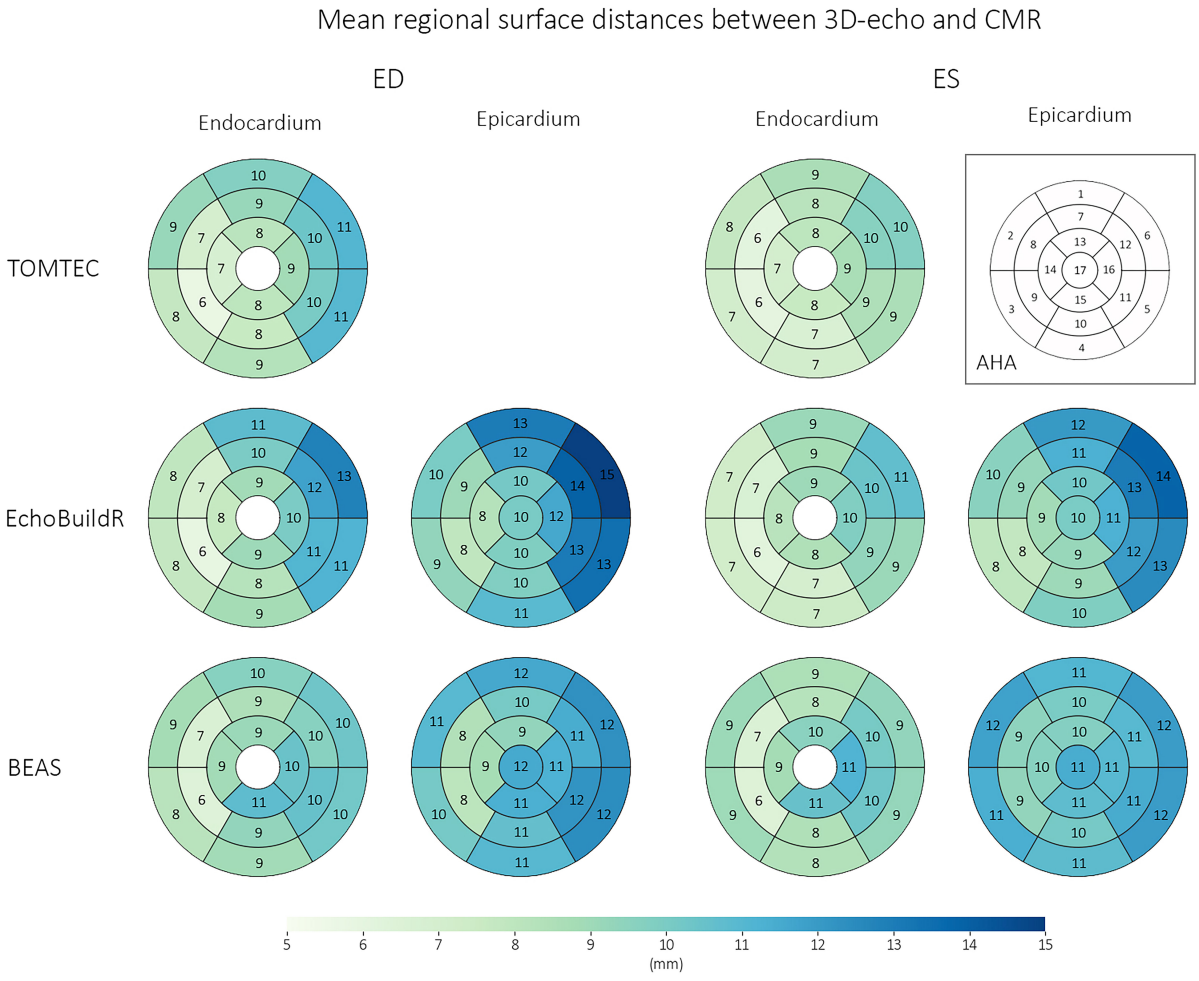
Regional surface distances (for the endocardium and epicardium) between corresponding 3D-echo and CMR models across *n* = 70 subjects, at ED and ES. Numbers represent mean surface distances in mm.

Following CMR and 3D-echo alignment, regional surface distances between corresponding models were evaluated (Figure 4). For all three 3D-echo analysis methods, the largest differences were found at the anterolateral regions (AHA segments 6 and 12) and toward the base at both ED and ES. Conversely, the smallest differences were observed at the mid-ventricle, toward the interventricular septum (AHA segments 8 and 9).

### Effect of Image Appearance

**Figure 5:**
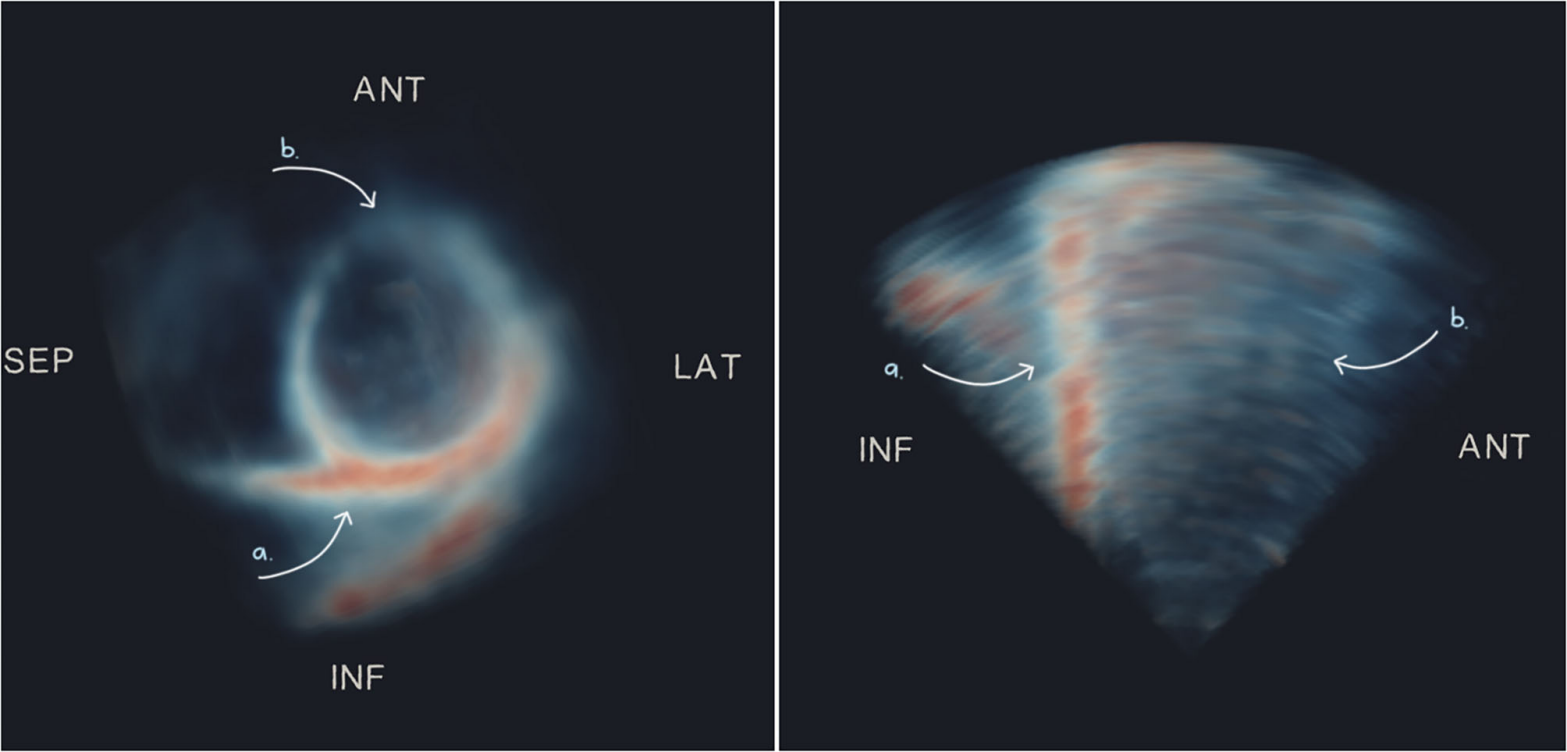
Maximum intensity projections of short- **(left)** and long- **(right)** axis views of an example 3D-echo image volume from a healthy participant, showing typical dropout on the anterior surface. Arrows indicate regions of high signal **(a)** and low signal or dropout **(b)**. ANT, anterior; LAT, lateral; SEP, septal; INF, inferior.

The appearance of the 3D-echo images was regionally heterogeneous, with systematic signal dropout generally occurring in the anterior (compared with inferior) regions, even in healthy participants (exemplified in Figure 5). Likewise, in patients with poor acoustic windows, the area of highest signal was found at the inferior surface (despite low visibility of all other wall segments). This was confirmed by calculating relative signal intensity as a percentage of the peak signal after normalization of intensity values between 0 and 95^th^ percentiles on a per-image basis. American Heart Association segment 10 (mid-inferior) had the highest mean signal intensity of 68%, compared to a mean signal intensity of 39% at segment 1 (basal-anterior), averaged across our study population.

A qualitative visual comparison of contours superimposed on images from each modality was performed to identify modality-dependent features that may have contributed to discrepancies between the constructed geometries. To create analogous views, 2D slices were extracted from the 3D-echo image volume coinciding with the CMR four- and two-chamber long-axis slices, as well as CMR short-axis slices.

**Figure 6:**
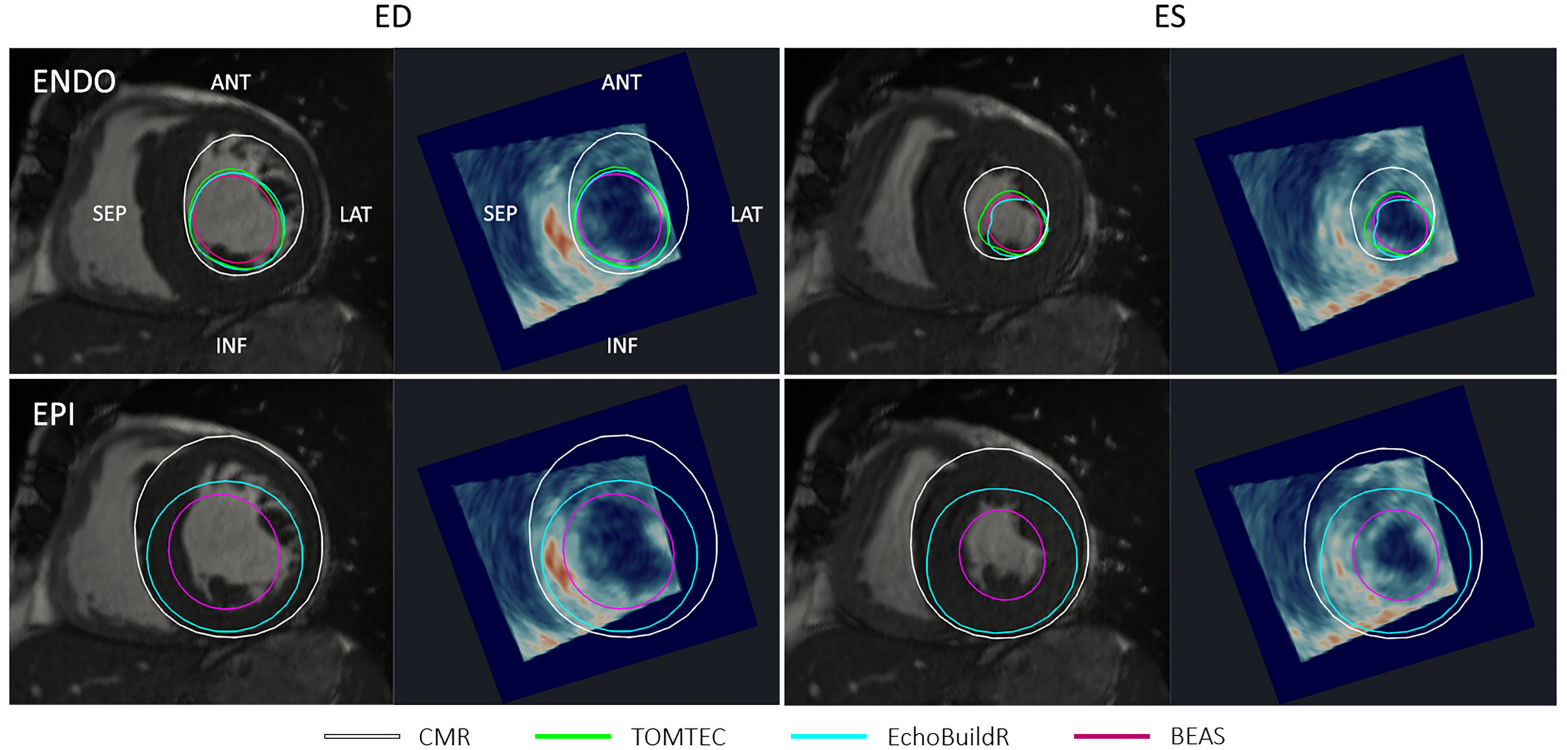
Short-axis CMR slice and corresponding resliced 3D-echo image at end-diastole (ED) and end-systole (ES) for a patient with hypertrophied myocardium. Top row shows endocardial (ENDO) contours, and bottom row shows epicardial (EPI) contours derived using each method indicated in the legend. ANT, anterior; LAT, lateral; SEP, septal; INF, inferior.

In areas with signal dropout or low tissue contrast, a larger discrepancy was observed between 3D-echo and CMR (as well as between the three 3D-echo image analyses). Additionally, the appearances of trabeculae at the LV lateral wall were often indistinguishable from the myocardium on 3D-echo—an effect that was more pronounced in the presence of LV hypertrophy (Figure 6). This effect also seemed to impact acquisition, whereby internal wall structures were mistaken for ventricular myocardium, resulting in the inadvertent exclusion of hypertrophied myocardium from the chosen FOV.

**Figure 7:**
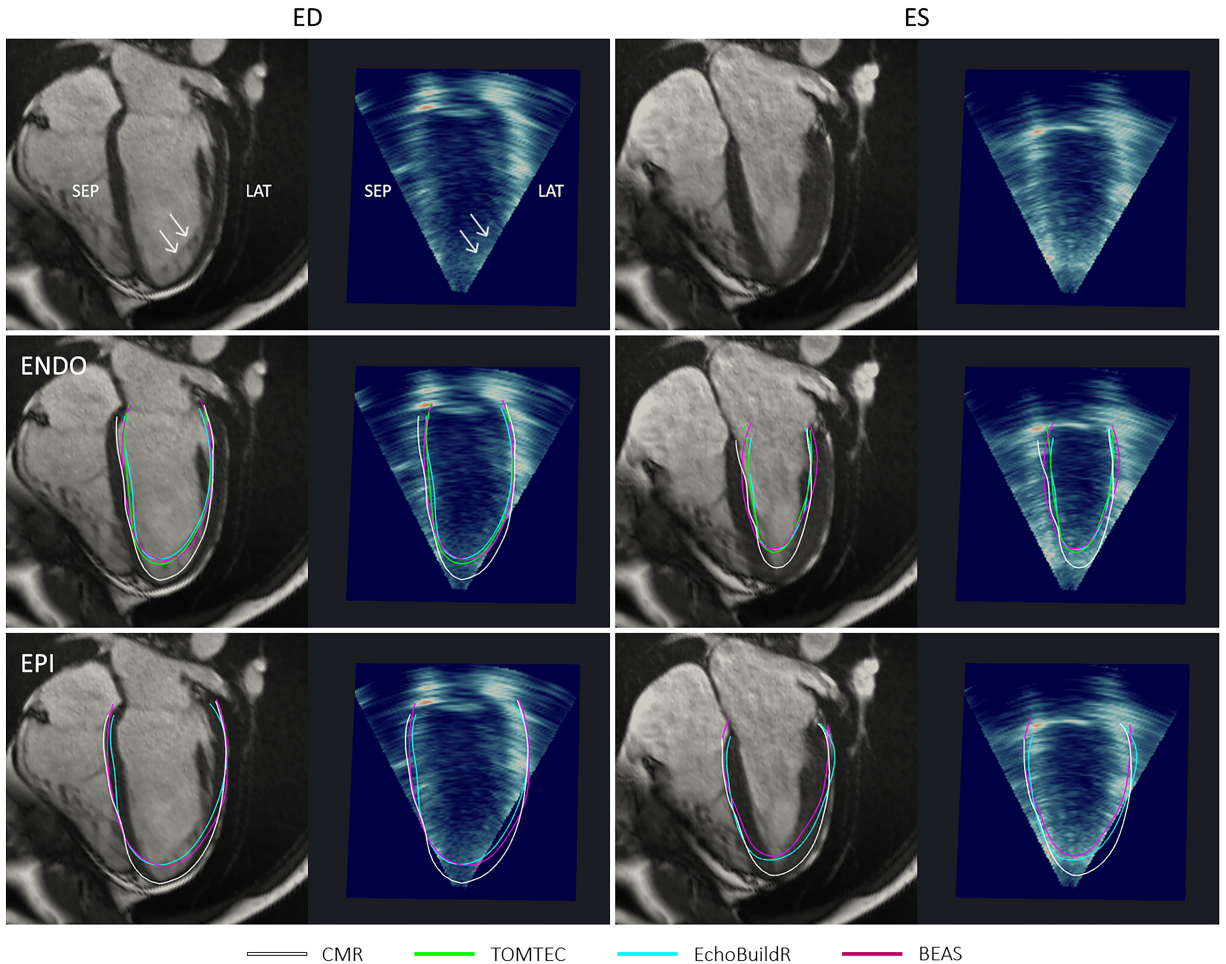
Four-chamber long-axis views by CMR and resliced 3D-echo from a healthy participant, at end-diastole (ED) and end-systole (ES). Top row indicates (with arrows) positions and appearances of apical trabeculations in each modality. Middle row shows endocardial (ENDO) contours, and bottom row shows epicardial (EPI) contours derived using each method indicated in the legend. ANT, anterior; LAT, lateral; SEP, septal; INF, inferior.

In the long-axis views, further discrepancies between 3D-echo and CMR myocardial contours were noted near the apex (Figure 7). On CMR images, apical trabeculations appeared as slight shadows, which could be distinguished from the myocardium. This distinction was less apparent on 3D-echo, leading to the illusion of a foreshortened apex. In addition to the absence of a clear apical endocardium, the apical epicardium was also obscured by its proximity to the thoracic wall. It was further observed that the LV cavity on 3D-echo appeared to be under-segmented at the inferior septum despite (and perhaps in part due to) the particularly bright signal, albeit to a lesser extent than that observed at the inferolateral wall.

At ES, there were larger variations between the endocardial surfaces generated using the three 3D-echo segmentation methods, although underestimation at the apex was still typically observed. Visually, there was no clear trend as to whether 3D-echo analyses were more likely to over or under-segment the LV cavity at ES.

### Reproducibility of 3D-Echo Models Across Software Tools

Intraclass correlation coefficients (as defined in section Statistics) were calculated to assess the reproducibility of LV models derived from 3D-echo using the TOMTEC, EchoBuildR, and BEAS methods. We found an ICC of 0.955 for global EDV; an ICC of 0.921 for ESV; and an ICC of 0.703 for LVM. This was supplemented by a combined Bland-Altman analysis of EDV and ESV (see Supplementary Figure 1), where the bias and 95% limits of agreement were calculated using the differences from each of the three methods to the mean of the measurements for each subject. Using this method, symmetric limits of agreement were ± 22 ml for EDV and ± 16 ml for ESV. The methods with the largest bias from the mean measurements were EchoBuildR for EDV (bias of 4 ml) and TOMTEC for ESV (bias of 7 ml).

### Inter-observer Variability for 3D-Echo and CMR Measurements

To account for inter-observer variability associated with each modality, ICCs were calculated for EDV and ESV between two observers, each on CMR and 3D-echo (TOMTEC). For CMR, the inter-observer ICC was 0.991 for EDV and 0.965 for ESV. In comparison, lower correlation scores were found for 3D-echo, with an inter-observer ICC of 0.872 for EDV and 0.803 for ESV.

**Figure 8:**
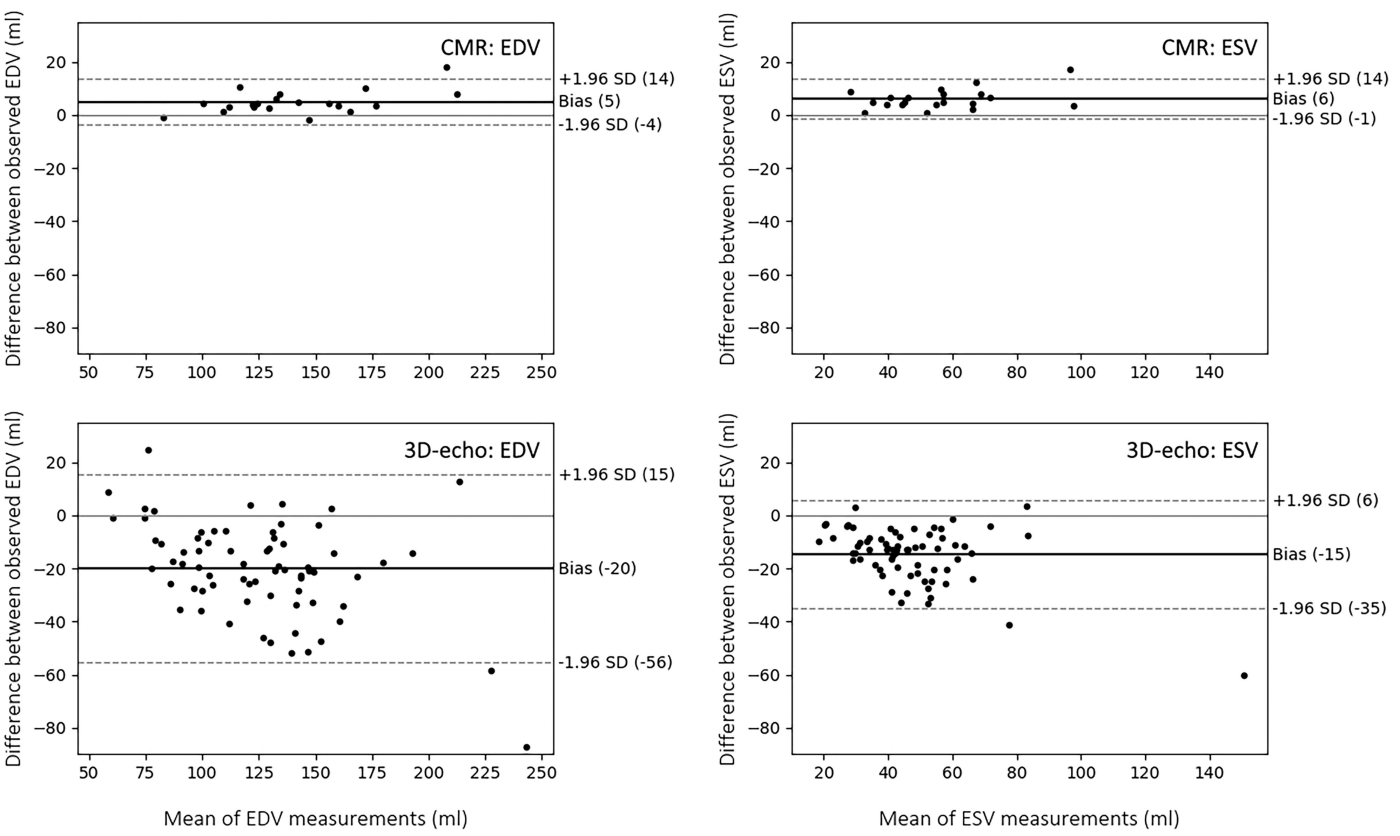
Bland-Altman plots showing limits of agreement and biases between two observers in the analysis of LV volume from CMR (using CIM) and 3D-echo (using TOMTEC). The limits of agreement represent the values between which 95% of the differences are expected to fall.

Bland-Altman analyses were performed to determine the limits of agreement for EDV and ESV between Experts A and B using CIM for CMR and between Experts A and C using TOMTEC for 3D-echo (Figure 8).

## Discussion

This study used multi-modality image fusion to investigate regional differences in LV geometry between 3D-echo and CMR. EDV was underestimated in 3D-echo, with the greatest differences in the anterolateral regions and the smallest differences in the inferoseptal regions. These differences were matched by regional heterogeneity in relative signal intensity and differences in the appearances of trabeculae.

Although there is a consensus that 3D-echo underestimates LV volume when compared to CMR (5), there remain large discrepancies between the magnitude of reported underestimations (expressed as mean ± 2 SD), ranging from −4 ± 43 ml (18) to −41 ± 37 ml (19) for EDV, and 0 ± 33 ml (18) to −34 ± 45 ml (20) for ESV. In this study group of 70 mixed subjects, the equivalent biases for EDV (−11 ± 50 ml to −18 ± 48 ml) and ESV (−1 ± 30 to −9 ± 36 ml) are within the ranges of those previously reported. For the semi-automatic methods (TOMTEC and EchoBuildR), volume underestimation was larger at ED than ES (as there were significant differences in EDV and not in ESV). From our own observations, as well as those described in the literature, trabeculations visible at ED are generally more difficult to differentiate from compact myocardium at ES when imaged by CMR (21, 22). Rather than an improved performance in 3D-echo analysis at ES, it seems more likely that ESV is underestimated using CMR due to inconsistency in analysis whereby trabeculae are included in the LV cavity at ED but subsequently excluded at ES. Such discrepancies in EDV and ESV are further propagated in terms of EF, which may consequently alter borderline functional diagnoses and eligibility for certain therapies. Indeed, statistically significant differences in EF were found between CMR and 3D-echo for all three analysis methods investigated in this study (Table 1). In contrast to EchoBuildR and TOMTEC, fully automatic methods such as BEAS are susceptible to failures (e.g., incorrect border detection) on edge cases, which potentially result in large volumetric errors and underperform semi-automatic methods. Nevertheless, correlation with CMR is expected to improve with manual verification and corrections in such circumstances.

We observed a systematic signal dropout during 3D-echo acquisition and subsequently found that on average, image regions corresponding to the inferior LV wall exhibited 74% brighter signal relative to image regions spanning the anterior wall. As a result, this may limit the accuracy of regional analyses (such as segmental strain, regional thickness or mass, and regional motion) for which the anterior aspect of the LV is concerned. This heterogeneity may be partly due to the anterior wall being most parallel to the ultrasound beam (in keeping with standard apical probe positioning), thus yielding poorer reflection. Previous studies have found similar patterns, resulting in the inferior RV insertion point assigned as the preferred landmark for short-axis LV orientation in 3D-echo (23). There are two potential approaches to redress the common anterior signal dropout. First, the functionality of 3D ultrasound systems during acquisition could be enhanced by applying spatially variable or adaptive gain in the direction of common dropout regions (to avoid over-gaining of the entire image). Second, post-processing in the analysis of such regions could be informed using a statistical shape template (14). In the absence of the proposed solutions above, it may be sensible to assume that a missing regional wall segment lies parallel to the ultrasound beam (as it is likely this configuration that elicits the dropout). While the implementations of these solutions are beyond the scope of the present study, future experiments may benefit from such solutions to achieve a higher degree of confidence when analyzing the anterior LV regions.

Trabeculae present on the LV lateral wall typically obscured the visibility of the endocardial boundary. Our analysis revealed that surface distances between the CMR and 3D-echo derived models were the largest in AHA segments 6 and 12 (on the anterolateral side), with average differences of up to 13 mm at the endocardium and 15 mm at the epicardium at ED. Quantitatively, the spatial distributions of surface distances for the 17 AHA regions were relatively consistent across the different methods for 3D-echo analysis (Figure 4), regardless of whether or not manual intervention was involved in the analysis.

Although there was generally high signal at the septal and inferior surfaces, under-segmentation of the LV cavity was still observed. One explanation is the relatively poor lateral resolution on 3D-echo (which further decreases toward the base), causing an apparent blurring between the myocardium and blood pool. This subsequently produced the appearance of a smaller cavity following the reconstruction of beamlines into a 3D cartesian image.

While it was not practical to isolate the individual factors (i.e., acquisition parameters and patient-specific acoustic properties) that affect 3D-echo image formation, we identified key features pertaining to differences between 3D-echo and CMR by a qualitative comparison of images. Typically, it is more difficult to visually distinguish between trabeculations and regular compact myocardium on 3D-echo than CMR. In most cases, the result is the misleading appearance of a reduced cavity volume (Figures 6, 7). While apical foreshortening can be mitigated in terms of acquisition by the transition from 2D to 3D imaging, our results show that this is not necessarily the case during analysis. In terms of the manual analysis of 3D-echo, agreement with CMR can be improved by using embedded models derived from corresponding CMR images (such as those produced in this study) during operator training. This enables the operator to become familiarize with the appearances of a reduced cavity volume resulting from low resolution 3D-echo by providing an objective reference, rather than relying on other human expertise, which may be subject to similar visual biases. For automatic solutions (as is the case for BEAS), algorithms may be refined to better correlate with CMR volumes by adjusting edge-detectors (e.g., gradient filters) such that resultant surfaces lie closer to the bright mid-myocardium.

It is well-established that both inter-observer variability and inter-software variability in 3D-echo analysis have significant effects on LV volumetric indices (24). In contrast to a previous study (25), our inter-observer variability was larger than inter-software variability, i.e., segmentations produced by the same observer using different software tools exhibited better agreement (higher ICC values, with a lower bias and narrower limits of agreement) than different observers using the same software. The observer with expertise in the analysis of both CMR and 3D-echo (Expert A) produced results with higher agreement between CMR and 3D-echo than the observer who had expertise in 3D-echo analysis alone (Expert C). Again, this suggests that operator training using both modalities could help reduce discrepancies between CMR and 3D-echo. Furthermore, image fusion provides a direct visual link between the two modalities to better understand discrepancies at the image level.

The higher agreement observed between the LV volumes estimated using the different software packages (ICC > 0.9 for EDV and ESV), compared to that of LVM (ICC ≈ 0.7), may have been partly due to software-specific volume rendering, which enhances the contrast between myocardium and blood pool, but decreases the identifiability of the epicardium. For this reason, the epicardial contour is typically set at a predefined radial distance from the endocardium in several 3D-echo analysis software packages (including EchoBuildR). This may bias the user when performing manual corrections, which becomes problematic in cases where the wall thickness is atypical (e.g., in hypertrophy) or asymmetric. Further advances in image quality are needed to improve agreement between 3D-echo analysis methods, as well as their correlation with CMR in terms of LVM.

Presently, as regional comparisons have been carried out for ED and ES geometries only, future analyses comparing CMR and 3D-echo across the entire heart cycle may provide an increased understanding of differences in regional motion.

### Study Limitations

Comparisons were carried out under the assumption that LV geometry is inherently identical under both CMR and 3D-echo, able to be rigidly aligned between acquisitions. Although multi-modal imaging was performed with minimal time between scans, images were nevertheless acquired at different instances, subject to physiological variability. Therefore, it may be more appropriate to register the images using a non-rigid transformation to account for the different body positions during CMR (supine) and 3D-echo (lateral).

The real-time (as opposed to gated) acquisition protocol used for 3D-echo was selected with region-specific modeling in mind, which can be complicated by the presence of stitching artifacts. In practice, gated acquisitions are more typically used for global volumetric assessment to maximize the spatial resolution for analysis, which consequently remains a trade-off in this study. The inclusion of additional ultrasound vendors as well as 3D-echo acquisition protocols may also enhance the generalizability of our findings.

Finally, the studied subjects consisted of a large proportion of healthy participants. Outcomes from this study may, therefore, need to be adjusted when analyzing purely clinical cases.

## Clinical Implications

For use of 3D-echo in a clinical context, care should be taken during both acquisition (to ensure an adequate FOV) and image analysis to avoid under-segmentation of the LV cavity or myocardium at anterior and lateral regions due to susceptibility to signal dropout and misleading appearances of trabeculae. Accordingly, clinical measurements pertaining to these regions, such as segmental strain or wall thickness, should also be interpreted with caution. Continuing research remains important to optimize the quantification of LV structure and function using 3D-echo, which will improve accuracy of echocardiographic assessment of the LV and serial measurements where needed.

## Data Availability Statement

The data underlying this article may be shared as part of the Cardiac Atlas Project (https://www.cardiacatlas.org/), upon application.

## Ethics Statement

The study protocol involving human participants was reviewed and approved by the Health and Disability Ethics Committee of New Zealand (reference 17/CEN/226). The patients/participants provided their written informed consent to participate in this study.

## Author Contributions

All authors have contributed significantly to the submitted work, including involvement in the research design, analysis and interpretation of data, and critical revision of the manuscript draft. The authors agree to be accountable for the content of the work.

## Funding

This study was funded by the Health Research Council of New Zealand (program 17/608 and project 17/234) and National Heart Foundation of New Zealand (project 1834), with support from the MedTech CoRE of New Zealand.

## Conflict of Interest

At study commencement, HH held a position as Advanced Development Product Manager at Siemens Healthineers and provided training toward the acquisition and analysis of 3D-echo data. JD'h currently holds research contracts with GE Vingmed. MN and AY held a research contract with Siemens Healthineers. MN was on the scientific advisory board for HeartLab NZ Ltd. The remaining authors declare that the research was conducted in the absence of any commercial or financial relationships that could be construed as a potential conflict of interest.

## Publisher's Note

All claims expressed in this article are solely those of the authors and do not necessarily represent those of their affiliated organizations, or those of the publisher, the editors and the reviewers. Any product that may be evaluated in this article, or claim that may be made by its manufacturer, is not guaranteed or endorsed by the publisher.

## Abbreviations

2D: two-dimensional
3D: three-dimensional
AHA: American Heart Association
BEAS: B-spline explicit active surfaces
CMR: cardiac magnetic resonance imaging
Echo: echocardiography
ED: end-diastole
EDV: end-diastolic volume
EDVI: end-diastolic volume index
ES: end-systole
ESV: end-systolic volume
ESVI: end-systolic volume index
FE: finite element
FOV: field of view
ICC: intraclass correlation coefficient
LV: left ventricle
LVM: left ventricular mass
LVMI: left ventricular mass index
MSD: mean surface distance
RV: right ventricle
SD: standard deviation
TR: repetition time
TE: echo time.
