# Supplementary material for: Systematic Comparison of Left Ventricular Geometry Between 3D-Echocardiography and Cardiac Magnetic Resonance Imaging
Source: Front Cardiovasc Med. 2021 Sep 20;8:728205. doi: 10.3389/fcvm.2021.728205 (PMC8488135; doi:10.3389/fcvm.2021.728205)
Supplement: Supplementary file 6 [file Data_Sheet_1.docx]

Supplementary Material

# Supplementary Figures


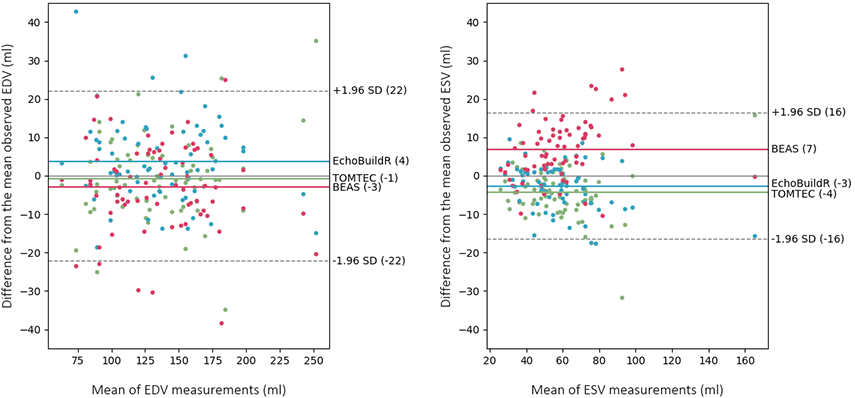


**Supplementary Figure 1.** Combined Bland-Altman analysis showing limits of agreement and biases for end-diastolic volume (EDV) and end-systolic volume (ESV) between three 3D-echo analysis methods (TOMTEC, EchoBuildR, and BEAS).

# Supplementary Videos

**Supplementary Video 1.** Visualization of rotation applied during manual alignment between CMR mesh and 3D-echo image.

**Supplementary Video 2.** Visualization of translation applied during manual alignment between CMR mesh and 3D-echo image.

**Supplementary Video 3.** Long-axis (4-chamber CMR) view of 3D-echo/CMR image fusion example from healthy volunteer (male, 23 years old).

**Supplementary Video 4.** Short-axis (mid-ventricular short-axis CMR) view of 3D-echo/CMR image fusion example from healthy volunteer (male, 45 years old).

**Supplementary Video 5.** Long-axis (3-chamber CMR) view of 3D-echo/CMR image fusion example from patient with LV hypertrophy (male, 54 years old).
